# Understanding adolescent health care services in Ghana: a scoping review

**DOI:** 10.11604/pamj.2024.48.179.40814

**Published:** 2024-08-15

**Authors:** Sheila Agyeiwaa Owusu, Allysa Warling, Joshua Arthur, Charles Martyn-Dickens, Anthony Enimil, Ransford Bio, Angela Osei-Bonsu, Leah Ratner

**Affiliations:** 1 Department of Pediatrics and Child Health, University for Development Studies, Tamale, Ghana,; 2 Department of Child Health, Komfo-Anoyke Teaching Hospital, Kumasi, Ghana,; 3 Department of Pediatrics and Child Health, Tamale Teaching Hospital, Tamale, Ghana,; 4 Harvard Medical School, Boston, Massachusetts, United States of America,; 5 Department of Public Health, Komfo-Anoyke Teaching Hospital, Kumasi, Ghana,; 6 Division of Global Health Equity, Brigham and Women’s Hospital, Boston, United States of America

**Keywords:** adolescent health, adolescent healthcare, adolescent care services, health services, young adult, developing countries, sexual and reproductive health, health policy, scoping review, Ghana

## Abstract

Over the last several decades, successful interventions in the health of newborns, infants, and children mean more children survive to become adolescents. There has been a global demand to improve health and care delivery for the adolescent population, guided by the United Nation’s Sustainable Development Goals by 2030. However, with this deadline fast approaching and with a rising adolescent population, this demand is ever more critical. Adolescent health requires a similar rights-based approach to ensure equitable distribution of healthcare interventions and service delivery going forward. This scoping review aims to explore the existing landscape of adolescent-responsive healthcare and service delivery in Ghana. It was conducted using the Joanna Briggs Institute (JBI) guidelines and reported according to the PRISMA-Scr standards. We searched the PubMed database from inception through May 2022 using the following search criteria: “Ghana” + “Adolescent” + “Health”. A total of 3172 studies were identified based on the search strategy outlined above, out of which 248 met the inclusion criteria. Both quantitative and qualitative analyses were conducted on all 248 studies to help synthesize findings. Overall, this review found that adolescent health care receives significant attention in Ghana, majority of which is focused on sexual and reproductive health (SRH). The studies available were a plethora of cross-sectional methods with large sample sizes, but their limited numbers of longitudinal studies and randomized control trials (RCTs) that could yield more robust evidence. This review is a call to action for a more comprehensive range of youth-driven, youth-responsive studies, interventions, and health programs that represent the whole range of challenges that confront adolescents in Ghana. This increased attention to adolescent needs will support a healthy cohort as they age into adulthood.

## Introduction

Adolescence marks the transition from childhood to adulthood and is typically defined as the second decade of life. It can be a challenging period with emotional, physical, and psychological changes. Adolescents constitute 1.2 billion of the world's population with more than two-thirds living in low or middle-income countries (LMICs). These figures are predicted to continue to increase over the next 35 years [1-3]. According to population estimates in 2020, nearly half of the population of Ghana (49%) were below the age of 20 and up to 22% were adolescents aged 10 to 19 (in their second decade of life) [4]. The United Nation’s Development Goals have fostered a critical demand to improve health and care delivery for this population by 2030 [5]. With this deadline fast approaching and this population growing, this need is ever more critical. Compared to other age groups, adolescents are at relatively low risk for overall morbidity and mortality and are therefore often overlooked. However, the WHO estimates that over 2000 preventable deaths occur daily amongst the adolescent cohort [2,3,6].

Globally, there has been a substantial push for policies and programs, to increase the survival of children under five. Over the last 30 years, there has been considerable success with survival in this age group. This is specifically illustrated by mass vaccination campaigns for vaccine-preventable diseases and the prevention of mother-to-child transmission of HIV such as mass vaccination campaigns for vaccine-preventable disease and the prevention of mother-to-child transmission of HIV (PMTCT), which have increased the survival of children under five. Over the last 30 years, there has been considerable success with survival in the under-5 age group. In Ghana specifically, there have been advancements in multi-sectoral programs supporting nutrition, malaria prevention and vaccination campaigns [4,7]. With this effective programming, more children are now surviving into adolescence, and thus need (and deserve) access to age-appropriate, rights-based health programming [8-10]. With the myriad and evolving health needs of the ever-growing population of adolescents, it is imperative that we start by documenting the current status of research, knowledge, policies, programs, and services which exist in Ghana. Scoping review methodology was chosen because of its exploratory approach so we could better understand how current research and knowledge are individually and collectively positioned to meet and address current challenges. The aim of this scoping review is to investigate this landscape of adolescent-specific healthcare policies, programs, and services, to address current and future needs.

## Methods

Taking an exploratory approach, this scoping review aims to identify and summarize scientific evidence on emerging questions, serving as a preliminary exercise for performing a future systematic review. We looked at literature encompassing both the causes and responses to adolescent-specific health care. This was intentionally broad in scope in order to understand the care needs and current care delivery capacity for this population in Ghana. An a priori protocol, which is available upon request, was developed through a consensus approach involving all members of the study team.

**Search strategy:** we searched the PubMed database from inception through May 2022 using the following search criteria: “Ghana” + “Adolescent” + “Health”. Given the type of scoping review and evidence sought, information sources were limited to PubMed-indexed publications. Grey literature was not included. Sources of evidence included studies that would help inform the current landscape of adolescent health programming. Case-control, cohort, randomized control studies, policy analysis, and qualitative and ethnographic studies were all included, as long as they met the inclusion criteria. Publications that were not population-focused, such as case studies or opinion pieces and editorials, were excluded.

We used these broad key terms with the aim of capturing work on both the prevalence of disease/illness in adolescent cohorts and adolescent-specific health services. Using the covidence platform, a group of reviewers (Leah Ratner, Sheila Agyeiwaa Owusu, Allysa Warling, Angela Osei-Bonsu, Joshua Arthur) screened titles and abstracts following inclusion and exclusion criteria which were developed as themes emerged. Each title was screened by two independent reviewers and then assessed for inter-rater reliability prior to advancing to full-text review. The extraction table (Annex 1) was created by the group and piloted prior to use.

**Eligibility criteria:** studies were included if they (i) focused exclusively on an adolescent age cohort (i.e., involved participants between ages 10-19; including studies that encompassed larger age ranges but had findings presented in a way that enabled filtering of the adolescent-specific age cohort) (ii) were conducted in Ghana (including studies encompassing other countries but with findings that could be stratified to Ghana), (iii) were published within the last 20 years (i.e., after 2003), and (iv) focused on a disease/illness, health care delivery, or health-related intervention (including peri and post-natal studies) and (iv) and were written in English. Of note, we also included studies of parents or caregivers of adolescents as long as they were focused on addressing adolescent healthcare needs. Studies were excluded if they; (i) were case studies or opinion pieces, (ii) had age cohorts that did not include adolescents and/or did not define an adolescent-specific age range in the results, (iii) were multi-country studies with results that could not be stratified to Ghana, or (iv) were related to antenatal care or other causes of adolescent morbidity and mortality that would not lend themselves well to adolescent-specific health care delivery, (v) were not full-text publications in English.

**Figure 1:**
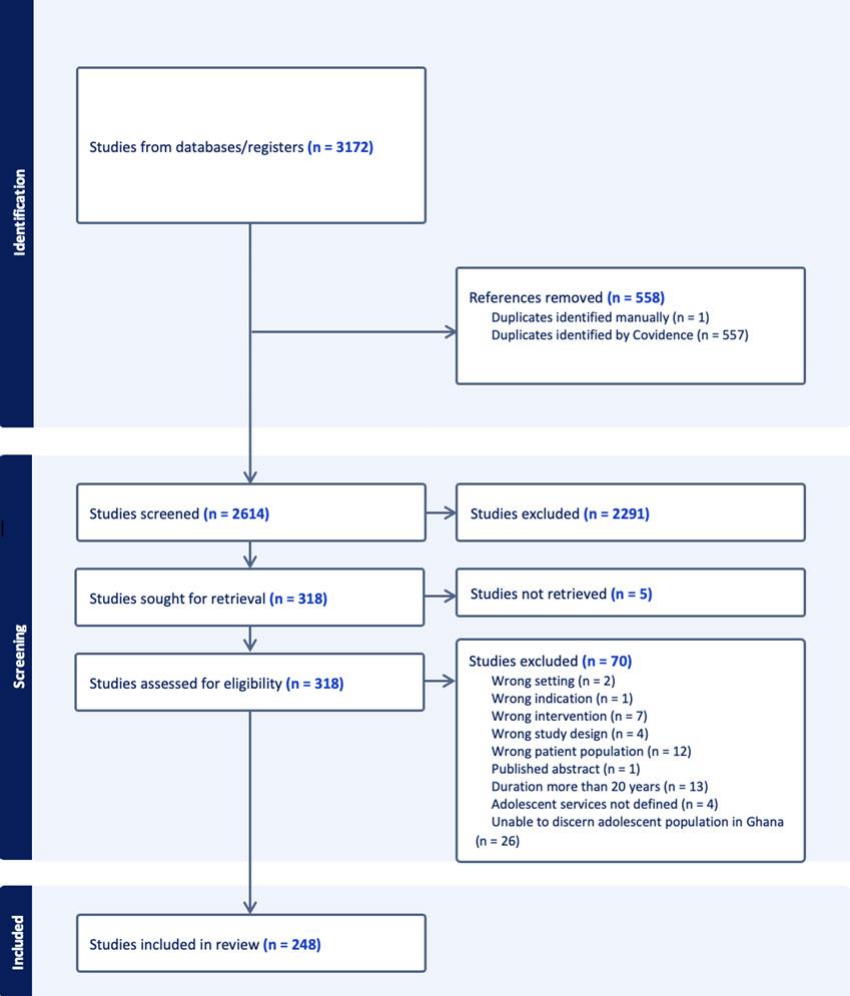
flowchart of study extraction, screening, and final inclusion

**Quality appraisal:** to assess and mitigate bias, each article was screened by two reviewers, and any disputes were resolved by a third reviewer. Potentially relevant articles were reviewed in full text and agreed upon by an independent second reviewer and then reviewed for inter-rater reliability. It should be noted, however, that only articles written in English were reviewed given this author group, which also allows for bias in the inclusion criteria favoring geographic areas (Figure 1).

**Quantitative analysis:** we quantitatively assessed and tracked several characteristics, including the percentage of Ghanaian authors on any given study, the study´s year of publication, the type of study, the age range and mean age of participants, the number of participants, and the sex breakdown of included participants (that is, whether a study focused on males, females, or both.) Assumptions were made that gender was reported only as binary (female or male) and therefore allows for bias for non-binary study participants (Annex 1). Data was cleaned and descriptive statistics were used to interpret each variable. Percentage of Ghanaian authors were manually counted. Mean and ranges were calculated using Microsoft Excel. Given this is a scoping review (and therefore exploratory) there was no indication for correlative statistical methods Table 1.

**Table 1:** summary of included studies

Number of included studies	% of Ghanaian authors	Study type (most often)	Mean year published	Mean age of participants	Mean N of participants	Study enrollment by gender
248	193 of 248 =77% (had at least one Ghanaian author)	Cross-sectional	2009	17.2 years	3210.28	67/248 only females, 2/248 only males

**Qualitative analysis:** given the exploratory nature of this scoping review, qualitative analysis was conducted for those variables that were not quantitative in nature. As part of the data extraction table, two independent reviewers recorded each study´s aim, outcomes, strengths, limitations, focus, and themes (Annex 1). The independent reviews were then imported into Taguette (taguette.org) and a coding dictionary was generated. Themes and study aim were coded with this same coding dictionary. This allowed for additional review to determine if the most common emerging themes were in alignment with the study aims. Coding yielded qualitative themes and, when appropriate, subthemes, for each category. The aims, outcomes, and themes sections of the data extraction table yielded nearly 100% saturation, so were collapsed into a single category of qualitative themes for analysis and visualization purposes.

**Current state of knowledge:** globally, the focus to date in adolescent health care has been on sexual and reproductive health (SRH) [5,10,11]. About 1 in 5 adolescent girls aged 15-19 years have unmet family planning needs, and approximately 7 million girls under the age of 18 conceive unintentionally every year [12-15]. This creates unnecessary social, financial, and psychological burdens on these adolescents, as well as increased vulnerability for medical complications. However, with Ghana´s ongoing demographic transition, its adolescent population is now faced with the double burden of communicable and non-communicable disease. This added dimension makes programming, research, and planning for health services challenging [16-18]. The breadth of issues facing the adolescent population is vast, with many preventable causes rooted in problems related to poverty. In addition, there is an emerging burden of chronic diseases amongst the adolescent cohort influenced by globalization including mental health concerns [19,20], as well as diseases associated with obesity and nutrient excess.

## Results

The initial search yielded 3127 results. The primary screen resulted in 318 articles, and full-text review yielded a final 248 articles. We included a total of 248 studies in our final quantitative and qualitative analyses (Figure 1).

**Figure 2:**
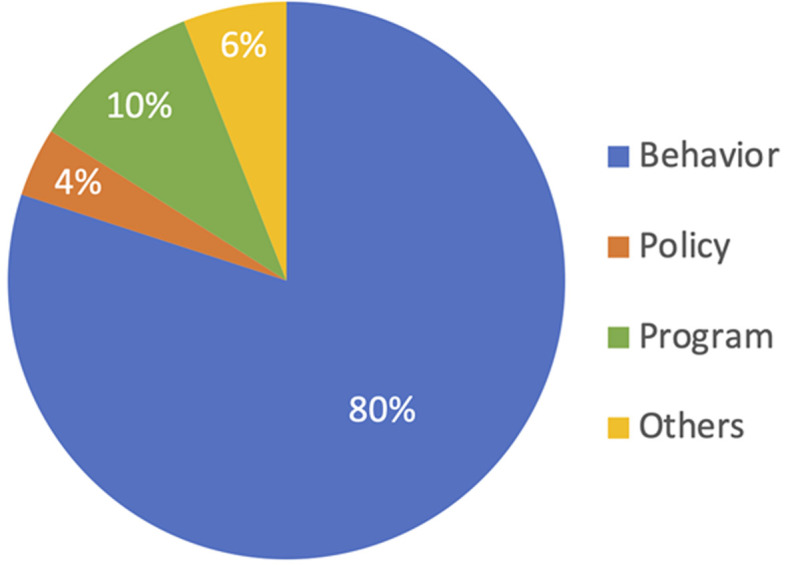
breakdown of areas studies addressed

**Quantitative findings:** Table 1 provides summary statistics for quantitative data. We also examined the focus of each study and found that the vast majority of studies (n= 200, or 80%) focused on an adolescent behavior or disease state, while 24 studies (10%) focused on specific programs related to adolescent health, and 9 studies (4%) focused on specific health policies related to adolescent health (Figure 2). Similarly, we found that 190 of the studies (~77%) focused on a cause of adolescent morbidity and mortality, while 58 (~23%) of studies focused on an adolescent-specific intervention(s). Taken together, these two findings suggest that most studies were focused on causes of adolescent morbidity and mortality, with far fewer studies focused on adolescent specific care delivery (i.e., interventions, programs, or policies). It is worth noting (Table 1), that the average N for studies reviewed was greater than 3000, which speaks to the robust nature of many of these studies with a large enrollment and good representation of the adolescent age group, as the average age enrolled was 17.2. There was a significant skew towards female only enrollment (27% or 67 studies) with only 2 studies (less than 1%) with male-only enrollment.

**Figure 3:**
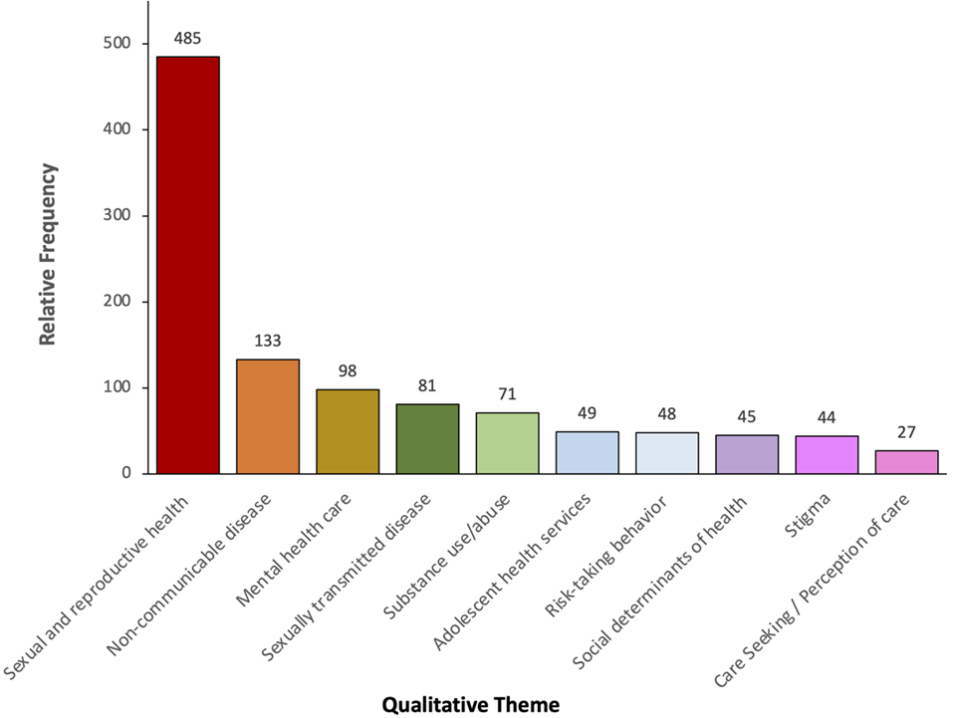
relative frequency of the top ten most common qualitative themes

**Qualitative findings:** Figure 3 shows the top ten most common qualitative themes, as well as their respective subthemes. The most common theme was sexual and reproductive health (with subthemes of generalized sexual and reproductive health, family planning, and adolescent pregnancy), followed by non-communicable diseases (including nutrition, physical activity, and studies on healthy weights for adolescents) and then mental health (including subthemes of generalized mental health and suicidal ideation). Additionally, the other themes included were sexually transmitted infections, substance use, adolescent health services, risk-taking behaviors, social determinants of health, stigma, and adolescent care seeking or perceptions of health care. The most common limitations noted were, that studies were not-generalizable, only examined correlations (and not causations), or that findings were subject to bias (noted to be most often self-report bias, recall bias, or social desirability bias). This was consistent with the quantitative results which showed that the majority of the studies we examined were cross-sectional, survey-based studies. The most common strengths were having a large sample size (consistent with quantitative findings above), being the first study in a marginalized group, and having nationally representative data. Of note, only few studies included a discussion of the study’s strengths, whilst most studies included a discussion of limitations. Limitations were derived from what was written in the study itself, not what was deemed a limitation by the study reviewer(s).

**Data visualization:** data insights were visualized utilizing Microsoft Excel and PowerPoint. Visualizations were created by one author (AW) and then independently reviewed by all authors to ensure they visually displayed correct synthesis.

## Discussion

Adolescence is a critical developmental period during which the provision of accessible, adaptable, and contextually appropriate care is crucial. Adolescents often under-utilize and under-seek care for a myriad of reasons [21-23]. Our aim was to better understand the landscape of adolescent-responsive health care in Ghana. It is worth noting that even though specific adolescent healthcare provisioning appears to be limited in Ghana and other places in Africa, there were a significant number of publications that met our inclusion criteria. Ghana continues to have robust research capacity and strong interest in publishing in this field, which is further illustrated by the large number of research articles returned by our search criteria as well as the large study sizes. The range of published research signals the presence of the unique characteristics, challenges and health needs of the adolescent population. In spite of this, our review showed there are still significant gaps in health service provision as well as low health knowledge and awareness levels amongst adolescents, themselves. First, many policies, interventions, and services focus on sexual and reproductive health suggesting a possible neglect of other important areas of adolescent health. As adolescent health practitioners in the US and Ghana, the relative paucity of programmatic and research attention being paid to other clinically significant areas such as nutrition [21], mental health [22], and chronic diseases was concerning. Second, the majority of the studies were cross-sectional in nature (56%) which is helpful for initial needs assessments but leaves gaps in knowledge around causality, correlation, and long-term associations of well-known factors and adolescent health indices. Third, similar to several other large reviews on the continent [23,24] there is a seeming absence of well-designed interventions with measurable, sustainable outcomes, even in well-funded areas like adolescent sexual and reproductive health.

**Study themes:** our study found a preponderance of services addressing reproductive health. This finding is similar to previously published reviews [22,25,26], which found a prominent focus on adolescent pregnancy and reproductive health. As shown by our thematic analysis and further reiterated by the number of studies that enrolled only female-identifying participants, aspects of SRH discussed in these studies included adolescent pregnancy, abortion care (both patient perception as well as adolescent responsive services), sexually transmitted infection(s), as well as a significant focus on HIV/AIDS in this population. HIV has continued to be a significant element of adolescent sexual and reproductive health in Ghana. Interestingly, Ghana has a lower proportion of HIV+ adolescents and young adults than other countries on the continent. However, we suspect the roots of this research and publishing focus are multifactorial, including remnants of large funding pushes directed by the Global North. However, while the value of research work done in this area is tremendous and has no doubt contributed to improvements in SRH for many Ghanaian adolescents, it is important to note the relative dearth of adolescent health research in many other equally but neglected areas fundamental to adolescent health and healthcare delivery. For example, as Ahinkorah BO *et al*. [24] indicates, there is a relative “absence of measurable time-bound initiatives” in this field of research.

The second most common theme was around non-communicable diseases and demonstrates the demographic transition that is most evident in this age group. Non-communicable diseases are important to consider for adolescents globally, especially for Ghana, which is a country at the intersection of extreme poverty and overabundance. These extremes have implications for critical but adolescent-sensitive approaches to lifestyle management and healthy eating habits. Therefore, it is reassuring that research on non-communicable diseases among adolescents features prominently in this scoping review. Although much less common compared to research on SRH, research on mental health and substance abuse were also common. Amongst these, tobacco use appeared to be a frequent sub-theme. Though outside the scope of this review, it appears that Ghana may have some unique influential factors driving tobacco uptake amongst adolescents [27]. It is possible that the emergence of the mental health theme ties in with Ghana's demographic transition as well as the global increase in awareness of, and attention to, both mental health issues and substance abuse.

**Types of studies:** given that adolescent health is relatively new as field of research, it makes sense that most of studies that met inclusion criteria, were cross-sectional in nature, and that there were too few superior study designs such as cohort studies and randomized controlled trials (RCTS). The most common theme in the limitations of these studies were that the results were not generalizable, followed by limited ability to determine causality. This is likely a product of a high prevalence of cross-sectional studies. Lastly, as demonstrated in Figure 2, the majority of the studies focused on causes of health issue rather than proposed interventions, which also correlates with the idea that this field remains nascent.

**Limitations:** given the exploratory design of a scoping review, which was aimed at uncovering and mapping broad topics, there is limited ability to confirm evidence or produce statements that guide decision-making. However, we still believe this scoping review yields important findings that can be used to further guide research, programming and policy. Secondly, through the exploration of this review, we found that there are a significant amount of non-Ghanaian authors publishing in the field. This author group wanted to comment on this as a possible limitation to truly understanding the needs of adolescent services in Ghana. However, we have noted a progressive trend that studies in recent years are more likely to have a larger percentage of Ghanaian lead authors. We feel it is important to recognize inherent colonial trends in publication patterns, for and by non-Africans, about Africa. There is a risk that those not familiar who are not familiar with the local context are not capturing the true essence of the issues that they seek to address. For this reason, we aimed to purposefully publish this work in an African journal that has its primary readership on the continent.

## Conclusion

Adolescent health and adolescent-specific health services are an important part of healthcare delivery and research. Whilst clearly considered important in Ghana, policy and programming needs for adolescent health can still improve, supported by more robust research to better understand the barriers and facilitators to health needs and health-seeking behaviors of this special population. Only then can health service delivery be targeted to achieve rights-based care for adolescents, leading off into healthier adulthood. Going forward, adolescent-responsive research and care delivery should be driven by Ghanaian adolescent voices. We advocate for well-designed, adolescent-centric longitudinal studies to shed more light on causality and correlation between well-known risk factors and the wide range of adolescent health concerns, not just of reproductive health issues. These interventions should be community-based and contextually derived. We also strongly advocate for integrative and collaborative care models, which have been shown to improve holistic care in these settings. In addition, it is critical that interventions targeting adolescents benefit from implementation science and robust evaluations adopting RCTs or other interventional methodologies, as practicable. Our scoping review is a call to action for community-based, youth-centric adolescent health services and research led by indigenous scientists, driven by and for the needs of the Ghanaian adolescent population.

### What is known about this topic

- *There has been substantial work completed over the past 20 years to support adolescent health in Ghana;*
- *Adolescence is a vulnerable developmental period, and much of the current research focus has been on sexual and reproductive health (SRH);*
- *As Ghana goes through a demographic transition, adolescents are experiencing new comorbidities that need to be addressed.*

### What this study adds

- *Although there have been robust efforts to publish relevant findings on adolescent programming, health services and research in Ghana, we have found current emphasis is weighted heavily on Sexual and Reproductive Health (SRH) leaving a significant gap in knowledge in other critical areas such as mental health and non-communicable diseases in this age group;*
- *Similarly, the majority of studies that were included in this review are cross-sectional, leaving a significant gap in longitudinal and cohort studies which could provide more generalizable results;*
- *Lastly, there is a gap in knowledge on how to improve adolescent-responsive programming and patient-first care in this population in Ghana.*

## Annex

**Annex 1**: detailed extraction sheet (PDF-240 KB)
